# BER and Channel Capacity Performance of an FSO Communication System over Atmospheric Turbulence with Different Types of Noise

**DOI:** 10.3390/s21103454

**Published:** 2021-05-15

**Authors:** Zixuan Xu, Guanjun Xu, Zhengqi Zheng

**Affiliations:** 1 Shanghai Key Laboratory of Multidimensional Information Processing, East China Normal University, Shanghai 200241, China; 51191214033@stu.ecnu.edu.cn; 2 Peng Cheng Laboratory, Shenzhen 518052, China; 3 Engineering Center of SHMEC for Space Information and GNSS, East China Normal University, Shanghai 200241, China; 4 School of Communication and Electronic Engineering, East China Normal University, Shanghai 200241, China; zqzheng@ee.ecnu.edu.cn

**Keywords:** free-space optical communication, noise, lognormal model, Gamma–Gamma channel, BER, channel capacity

## Abstract

The propagation performance of a free-space optical (FSO) communication system in an atmospheric environment is restricted and degraded due to the influence of atmospheric turbulence. In this paper, both the lognormal and Gamma–Gamma channel models are employed to characterize this turbulence under weak-to-strong conditions. In addition, the average bit error rate and average channel capacity of an FSO communication system under the influence of background noise, thermal noise and quantum noise (resulting from the environment, the device, manual operation, etc.) are considered. Moreover, the comparison of system performance under different turbulence conditions and various noises are conducted. Simulation results reveal that thermal noise has a dominant effect on the FSO system. In addition, both the channel parameters and the system parameters have a significant influence on the performance of an FSO communication system.

## 1. Introduction

Free-space optical (FSO) communication has attracted considerable attention due to its advantages of lower cost, higher data rates, and higher security for many wireless communication applications [1,2]. FSO communication is therefore considered a promising technology that will play a significant role in research on fifth-generation and even sixth-generation communication [3,4]. However, an FSO communication system is hampered by several challenges when an optical wave propagates in atmospheric turbulence [5]. The intensity of the optical signal fluctuates during this period, in a process referred to as scintillation, since the refractive index of the atmosphere turbulence changes randomly as a result of variations in the atmospheric temperature and atmospheric pressure [6,7].

Recently, several probability distribution functions (PDFs) have been proposed for various channel models to characterize the fading process of the optical signal in atmospheric turbulence. For turbulence under weak condition, the lognormal fading model is employed, as the signal obeys a lognormal distribution [8,9]. In view of the influence of atmospheric turbulence under the weak conditions on an optical signal, the performance metrics of the FSO system with maximum-likelihood detection are investigated under the weak turbulence with a lognormal model [10].

In addition, a negative exponential model has been proposed, and has been shown to be a suitable model for strong and even saturation scintillation [11]. In [12], the negative exponential distribution was employed to model FSO links with fading statistics, and the outage probability and the average capacity of the communication system were studied under some limited conditions for the practical applications. Derived from the lognormal model, the *K* turbulence channel model has also been proposed for strong turbulence conditions [13]. A study of the propagation of laser light with *K* distribution was carried out in [14,15]. In addition, Kumar et al. proposed exact expressions for the bit error rate (BER) and channel capacity for an FSO communication system under both lognormal and *K* distribution conditions. In order to investigate atmospheric turbulence under moderate-to-strong conditions, the Gamma–Gamma distribution was proposed in [16]. The performance of an FSO system in terms of the outage probability, average BER, and ergodic capacity in various communication scenarios, such as an unmanned aerial vehicle (UAV)-based optical communication, inter-satellite/satellite-ground laser communication, and underwater optical communication, has been extensively studied [17,18,19].

In addition to atmospheric turbulence, noise (such as thermal noise, background noise, quantum noise, etc.) also has a significant influence on the FSO communication system. During the transmission process with high symbol rates, heating emerges on the device [20,21]. Therefore, a variation in temperature can be produced, giving rise to thermal noise [22,23]. Due to the wave-particle duality of optical signals, this type of transmission is also called quantum transmission. At the receiving end, a photodetector receives these quanta of light. The change in phase arising from various factors such as jitter at the detector results in a decline in the number of photons received, an effect which is known as quantum noise [24]. In addition to effects created by devices, background noise from manual operation and environmental noise also need to be considered [25].

Recently, several works have investigated the influence of various types of noise on FSO communication system. For instance, the effects of both an avalanche photodiode (APD) and thermal noise on an optical system with binary phase-shift keying (BPSK) subcarrier-intensity modulation over weak-to-strong turbulence channels are discussed in [6]. The effects of both pointing errors and phase noise on the terrestrial-FSO link have also been studied for a subcarrier phase-shift keying (PSK) modulation scheme [26]. The authors in [27] investigated the average symbol error rate of M-ary PSK modulation over various fading channels, which is also subject to phase noise. In [22], the influence of thermal noise, shot noise, and background noise on the performance of P-i-N (PIN) diodes and APD-based FSO communication system was analyzed under weak turbulence conditions. However, to the best of our knowledge, the influence of various types of noises on the FSO communication system are rarely studied. Moreover, the study on the comparison of system performance under different turbulence conditions influenced by several types of noise has also seldom been investigated.

Motivated by the above analysis, to fill this gap, the impacts of thermal noise, quantum noise, and background noise on the average BER and average capacity of an FSO communication system are investigated. Besides, a detailed comparison of the system performance under various turbulence conditions is discussed in this study. We also explore the improvements in system performance from the use of different modulation schemes.

The remainder of this paper is organized as follows. In Section 2, the system and channel models are introduced in order to analyze the processes in the FSO communication system. Exact closed-form results for the average BER and the average channel capacity are derived in Section 3, taking into consideration thermal noise, quantum noise, and background noise. Simulation results and the discussion are presented in Section 4, followed by the conclusions in Section 5.

## 2. System and Channel Models

**Figure 1:**
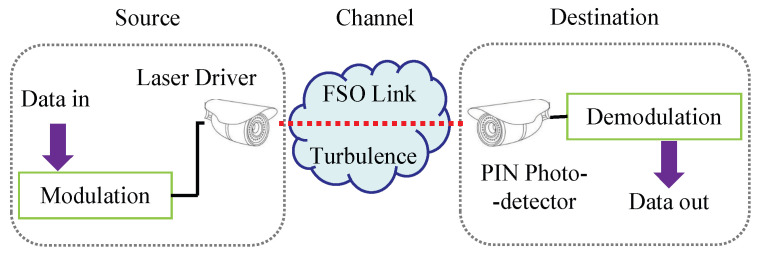
System model for optical wave propagation in atmospheric turbulence.

A diagram of an FSO communication system with modulation and demodulation schemes is shown in Figure 1. The electrical signal is first modulated using the modulation scheme, and then converted into an optical signal by a laser driver. Following this, the optical signal is transmitted to the receiver through atmospheric turbulence. Refraction, reflection, scattering and other influences arising from the variation in the refractive index of the turbulence environment result in fluctuations in the phase and amplitude, referred to as scintillation. The received signal is then converted into an electrical signal by a PIN photodetector and finally demodulated using the corresponding demodulation model. The signal after demodulation can be written as

(1) $$y=IRA\xi m\left(t\right)+n\left(t\right),$$

where *R* is the responsivity of the photodetector, *I* is the half peak of the light intensity, and ξ is the modulation index. In addition, *A* is the amplitude of the subcarrier, *m*(*t*) and *n*(*t*) are the electrical signal and the additive white Gaussian noise subject to a distribution *N*(0, $\sigma^{2}$), respectively, and $\sigma^{2}$ is the noise variance.

In order to give a comprehensive analysis of the influence of different types of noise on the communication system, we consider background noise, thermal noise and quantum noise in this study. Hence, the total noise is expressed as

(2) $$\sigma^{2}=\sigma_{bg}^{2}+\sigma_{T_{e}}^{2}+\sigma_{Quantum}^{2},$$

where $\sigma_{bg}^{2}$, $\sigma_{T_{e}}^{2}$, $\sigma_{Quantum}^{2}$ denote the background noise, thermal noise, and quantum noise, respectively. Note that the above-mentioned forms of noise can also be expressed as, $\sigma_{bg}^{2}=\frac{2qI_{bg}R_{b}}{RI_{o}^{2}}$, $\sigma_{T_{e}}^{2}=\frac{4k_{1}T_{e}R_{b}}{RR_{L}I_{o}^{2}}$, $\sigma_{Quantum}^{2}=\frac{2qR_{b}}{RI_{o}}$ [28], where *q* is the elementary charge, $I_{bg}$ is the background irradiance, $R_{b}$ is the symbol rate, $I_{o}$ is average received irradiance, $k_{1}$ is the Boltzmann constant, $T_{e}$ is the temperature and $R_{L}$ is the load resistance of the receiver circuit. Based on the signal intensity and noise, the signal-to-noise ratio (SNR) can be given as $SNR=\frac{{\left(IRA\xi \right)}^{2}P_{m}}{\sigma^{2}}$, where $P_{m}$ is the subcarrier signal power [11].

As mentioned above, the optical signal will be scattered as it passes through atmospheric turbulence. For the case of different weather conditions in the transmission link, a lognormal distribution model is employed to characterize the atmospheric turbulence under the weak regime in this study. The Gamma–Gamma distribution model is also introduced to characterize the turbulent channel, as it has been shown to give high accuracy under moderate-to-strong conditions.

### 2.1. Lognormal Distribution Model for Weak Turbulence

According to [13], with respect to *I*, the probability density function (PDF) of a lognormal distribution can be expressed by

(3) $$p\left(I\right)=\frac{1}{I\sqrt{2\pi }\sigma_{x}^{2}}\exp\left(-\frac{{\left(\ln I+\frac{\sigma_{x}^{2}}{2}\right)}^{2}}{2\sigma_{x}^{2}}\right),$$

where $\sigma_{x}^{2}$ is the log irradiance variance, which can be written as

(4) $$\sigma_{x}^{2}=1.23C_{n}^{2}k^{\frac{7}{6}}L^{\frac{11}{6}},$$

where $C_{n}^{2}$, *k*, and *L* are the structure constants of the atmospheric turbulence, wave number, and communication distance, respectively.

### 2.2. Gamma-Gamma Distribution Model for Moderate-to-Strong Turbulence

Apart from the weak turbulence, the communication link will also encounter moderate-to-strong turbulence. Therefore, the moderate-to-strong turbulence, which can be characterized by the Gamma-Gamma distribution model, is also introduced to compare the system performance under various noises and the results of the comparison are presented in Section 4. In the Gamma–Gamma distribution model, the influence of both large-scale and small-scale turbulence on the optical signal is considered, and is assumed to obey a Gamma distribution. The PDF of the optical signal scattered by the atmospheric turbulence under moderate-to-strong conditions can therefore be expressed as [3],

(5) $$p\left(I\right)=\frac{2{\left(\alpha \beta \right)}^{\frac{\alpha +\beta }{2}}}{\Gamma \left(\alpha \right)\Gamma \left(\beta \right)}I^{\frac{\alpha +\beta }{2}-1}K_{\alpha -\beta }\left(2\sqrt{\alpha \beta I}\right),$$

where $\Gamma \left(\cdot \right)$ and $K_{n}\left(\cdot \right)$ are the Gamma function and a Bessel function of the second kind and in the *n*-th order, respectively. α and β are the effective turbulence numbers at small and large scales, and are given as

(6) $$\alpha ={\left[\exp\left(\frac{0.49\sigma_{x}^{2}}{{\left(1+1.11\sigma_{x}^{\frac{12}{5}}\right)}^{\frac{7}{6}}}\right)-1\right]}^{-1},\beta ={\left[\exp\left(\frac{0.51\sigma_{x}^{2}}{{\left(1+1.11\sigma_{x}^{\frac{12}{5}}\right)}^{\frac{5}{6}}}\right)-1\right]}^{-1}.$$

## 3. System Performance under Both Weak Turbulence and Moderate-to-Strong Turbulence

In this section, closed-form expressions are derived for the average BER and the average capacity of an FSO communication system under lognormal and Gamma–Gamma channel turbulence.

The average BER of the FSO communication system can be expressed as

(7) $$P_{e}=\int_{0}^{\infty }P_{ber}p\left(I\right)dI,$$

where $P_{ber}$ is the BER of the FSO communication system with BPSK modulation and coherent detection, given by [11]

(8) $$P_{ber}=\int_{0}^{\infty }\frac{1}{\sqrt{\pi }\sigma }\exp\left[-\frac{\left(i_{d}+0.5RIA\xi \right)}{\sigma^{2}}\right]di_{d}=Q\left(\frac{RIA\xi }{\sqrt{2\sigma^{2}}}\right),$$

where $Q\left(\cdot \right)$ is the *Q*-function from [13] with $Q\left(x\right)=\frac{1}{2\sqrt{\pi }}G_{1,2}^{2,0}\left[\frac{x^{2}}{2}\left|\begin{array}{c} 1\\ 0,\frac{1}{2} \end{array}\right.\right]$, where $G_{p,q}^{m,n}\left(\left|\begin{array}{c} \cdots \\ \cdots \end{array}\right.\right)$ is the Meijer’s G function and the definition is seen in Appendix A. On the other hand, $\sigma^{2}$ in Equation (8) is the total noise including background noise, thermal noise, and quantum noise. In addition, $i_{d}$ is the baseband signal, and can be expressed as

(9) $$i_{d}\left(t\right)=\frac{d_{j}RIA\xi }{2}+n_{d}\left(t\right),$$

where $d_{j}$ is −1 or 1 for the *j*-th data packet representing the symbol for “0” or “1” and $n_{d}$(*t*) is the Gaussian white noise, subject to a distribution of $N(0,\sigma^{2})$.

Another parameter that is crucial in characterizing the performance of an FSO communication system is the average channel capacity, which refers to the maximum information transmitted per unit time, and this is also evaluated here. According to Shannon’s theorem, the channel capacity under invariant optical intensity can be expressed as $C=B\log_{2}\left(1+SNR\right)$.

For a FSO communication system under a specific turbulence condition, the average channel capacity under the corresponding channel model can be expressed as

(10) $$C=\int_{0}^{\infty }B\log_{2}\left(1+SNR\right)p\left(I\right)dI.$$

### 3.1. Average BER and Channel Capacity under Weak Turbulence

To analyze the system performance influenced by various noises under weak turbulence condition, the expressions for the average BER and average capacity are derived. Following the approach detailed in [29], *Q*-function can be expressed as $Q\left(t\right)=\frac{1}{\pi }\int_{0}^{\frac{\pi }{2}}\exp\left(-\frac{t^{2}}{2\sin^{2}\left(\theta \right)}\right)d\theta$, *x* > 0. Then, according to Gauss-Hermite polynomials, the integration is approximately written as $\int_{-\infty }^{\infty }f\left(x\right)\exp\left(-x^{2}\right)dx\approx \sum_{i=1}^{n}w_{i}f\left(x_{i}\right)$, where $w_{i}$ and $x_{i}$ are the weights and zeros of the Hermite polynomials [29]. Finally, an expression for the average BER under weak turbulence can be derived as

(11) $$P_{e}=\frac{1}{2\sqrt{\pi }}\sum_{i=1}^{n}w_{i}\mathrm{erfc}\left(\sqrt{\frac{\gamma }{4}}\exp\left(\sqrt{2}\sigma_{x}x_{i}-\frac{\sigma_{x}^{2}}{2}\right)\right),$$

where $\sigma_{x}^{2}$ is the log irradiance variance, γ is the normalized SNR expressed as $\gamma =\frac{R^{2}A^{2}}{\sigma^{2}}$, $\sigma^{2}$ denotes the total noise which has a significant impact on average BER and has been explained in Equation (8), and $\mathrm{erfc}\left(\cdot \right)$ is the complementary error function defined as $\mathrm{erfc}\left(x\right)=\frac{2}{\sqrt{\pi }}\int_{x}^{\infty }\exp\left(-t^{2}\right)dt$.

Note that the average BER is related to the log irradiance variance and the normalized SNR. In addition, structural constants, number of waves, binding distance result in log irradiance variance and total noise results in normalized SNR. Therefore, the average BER influenced by structure constants, link distance, and total noise is studied in this study.

For weak turbulence, the average channel capacity *C* can be obtained by substituting Equation (3) to Equation (10). After some mathematical manipulations, a closed-form expression for the channel capacity under weak turbulence can be achieved as [28]

(12) $$C=\frac{B}{\sqrt{\pi }}\sum_{i=1}^{n}w_{i}\log_{2}\left(1+\gamma \exp\left(2\sqrt{2}\sigma_{x}x_{i}-\sigma_{x}^{2}\right)\right),$$

where *B* is the bandwidth.

### 3.2. Average BER and Channel Capacity under Moderate-to-Strong Turbulence

Here, we further investigate the average BER and channel capacity of the communication system under moderate-to-strong turbulence, which is modeled by Gamma–Gamma distribution as we have mentioned in Section 2.

For moderate-to-strong turbulence, by inserting Equations (5) and (8) into Equation (7), the average BER can be written as

(13) $$P_{e}=\int_{0}^{\infty }Q\left(\frac{RAI}{\sqrt{2}\sigma }\right)\frac{2{\left(\alpha \beta \right)}^{\frac{\alpha +\beta }{2}}}{\Gamma \left(\alpha \right)\Gamma \left(\beta \right)}I^{\frac{\alpha +\beta }{2}-1}K_{\alpha -\beta }\left(2\sqrt{\alpha \beta I}\right)dI.$$

Note that the Bessel function in Equation (13) can be further recast with Meijer’s G function from [29] as

(14) $$K_{v}\left(x\right)=\frac{1}{2}G_{0,2}^{2,0}\left[\frac{x^{2}}{4}\left|\begin{array}{c} -\\ \frac{v}{2},-\frac{v}{2} \end{array}\right.\right].$$

By substituting the *Q*-function and Bessel function expressed by Meijer’s G function into Equation (13), the average BER can be written as

(15) $$P_{e}=\int_{0}^{\infty }\frac{{\left(\alpha \beta \right)}^{\frac{\alpha +\beta }{2}}}{2\sqrt{\pi }\Gamma \left(\alpha \right)\Gamma \left(\beta \right)}G_{0,2}^{2,0}\left[\frac{\gamma I^{2}}{4}\left|\begin{array}{c} 1\\ 0,\frac{1}{2} \end{array}\right.\right]G_{0,2}^{2,0}\left[\alpha \beta I\left|\begin{array}{c} 1\\ \frac{\alpha -\beta }{2},\frac{\beta -\alpha }{2} \end{array}\right.\right]dI.$$

Based on the properties of Meijer’s G function [30], the average BER for an FSO communication system under moderate-to-strong turbulence can be finally simplified as

(16) $$P_{e}=\frac{2^{\alpha +\beta -3}}{\pi^{\frac{3}{2}}\Gamma \left(\alpha \right)\Gamma \left(\beta \right)}G_{5,2}^{2,4}\left[\frac{4\gamma }{{\left(\alpha \beta \right)}^{2}}\left|\begin{array}{c} \frac{2-\alpha }{2},\frac{1-\alpha }{2},\frac{2-\beta }{2},\frac{1-\beta }{2},1\\ 0,\frac{1}{2} \end{array}\right.\right].$$

By substituting Equation (5) into Equation (10), the average channel capacity of the FSO communication system under moderate-to-strong turbulence can be expressed as

(17) $$C=\int_{0}^{\infty }B\log_{2}\left(1+\gamma I^{2}\right)\frac{2{\left(\alpha \beta \right)}^{\frac{\alpha +\beta }{2}}}{\Gamma \left(\alpha \right)\Gamma \left(\beta \right)}I^{\frac{\alpha +\beta }{2}-1}K_{\alpha -\beta }\left(2\sqrt{\alpha \beta I}\right)dI.$$

With the aid of the identical equations in Equation (14) and $\log_{2}\left(1+x\right)=\frac{1}{\ln2}G_{2,2}^{1,2}\left[x\left|\begin{array}{c} 1,1\\ 1,0 \end{array}\right.\right]$, we have

(18) $$C=\int_{0}^{\infty }\frac{B{\left(\alpha \beta \right)}^{\frac{\alpha +\beta }{2}}}{\ln2\Gamma \left(\alpha \right)\Gamma \left(\beta \right)}I^{\frac{\alpha +\beta }{2}-1}G_{2,2}^{1,2}\left[\gamma I^{2}\left|\begin{array}{c} 1,1\\ 1,0 \end{array}\right.\right]G_{0,2}^{2,0}\left[\alpha \beta I\left|\begin{array}{c} -\\ \frac{\alpha -\beta }{2},\frac{\beta -\alpha }{2} \end{array}\right.\right]dI.$$

Finally, the average channel capacity of the FSO system under Gamma-Gamma channel turbulence can be simplified with the aid of the integration property of Meijer’s G function [30] as

(19) $$C=\frac{B2^{\alpha +\beta -2}}{\pi \ln2\Gamma \left(\alpha \right)\Gamma \left(\beta \right)}G_{6,2}^{1,6}\left[\frac{16\gamma }{{\left(\alpha \beta \right)}^{2}}\left|\begin{array}{c} 1,1,\frac{1-\alpha }{2},\frac{2-\alpha }{2},\frac{1-\beta }{2},\frac{2-\beta }{2}\\ 1,0 \end{array}\right.\right].$$

## 4. Simulations and Discussion

In this section, the effects of the system parameters and channel parameters on the performance of the FSO communication system are analyzed. The relevant parameters used for the simulation are shown in Table 1. Note that the value of background radiation $I_{bg}$ in Table 1 is considered, including sky irradiation and Sun irradiation with a wavelength of 850 nm and an optical filter bandwidth of $10^{-3}$ μm, which are selected from [13,28]. The results of the simulations for both the average BER and the average channel capacity of the FSO communication system are given below. Meanwhile, the accuracy of the analytical expressions is corroborated by Monte Carlo (MC) simulations using over $10^{6}$ independent runs.

**Figure 2:**
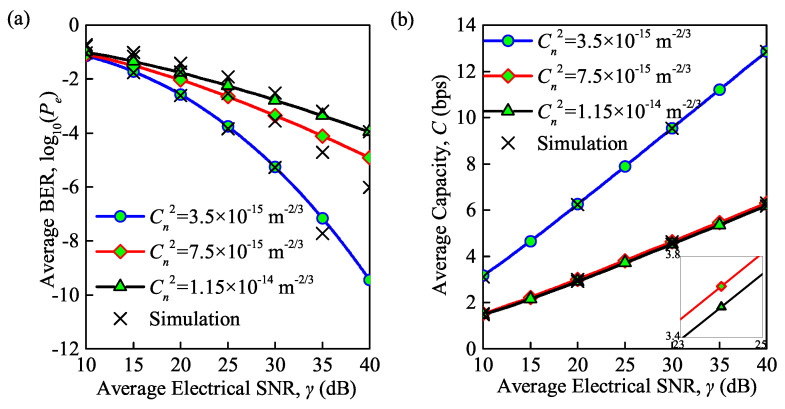
(**a**) Average BER versus normalized SNR for different structure constants; (**b**) average capacity versus normalized SNR for different structure constants (λ = 850 nm, $T_{e}$ = 300 K, $R_{b}$ = 0.155 Gb/s).

The simulation and analytical results of the system performance in terms of the average BER and average channel capacity, as influenced by the structure constant and average electrical SNR, is shown in Figure 2a,b, respectively. Note that the average BER in Figure 2a is based on Equations (11) and (16) for the case of both weak turbulence and moderate-to-strong turbulence, while the average capacity in Figure 2b is based on Equations (12) and (19). Note that similar simulation also has been conducted in [13,28] but without considering the effect of the total noise. To fill this gap, the total noise, which is comprised by the background noise, thermal noise, and quantum noise as shown in (2), is considered in the following simulation unless otherwise specified. The accuracy of the derived analytical channel model which depends on average electrical SNR can be observed. The structure constants were $0.35\times 10^{-14}$, $0.75\times 10^{-14}$ and $1.15\times 10^{-14}$$m^{-2/3}$, corresponding to weak, moderate and strong turbulence, respectively. The transmission channel under weak turbulence was simulated using a lognormal model, while the Gamma–Gamma model was used to describe the transmission channel under moderate-to-strong turbulence. With an increase in the average electrical SNR, a lower average BER and higher average capacity can be achieved. For a larger structure constant representing a stronger turbulence regime, the average BER is higher and the average capacity is smaller. This phenomenon can be explained by the fact that the strength of the atmospheric turbulence is directly determined by the structure constant. The Rytov variance for the propagation of an optical wave in atmospheric turbulence becomes larger with an increase in the structure constant, and finally results in larger average BER and smaller average channel capacity for the FSO communication system. These effects change slowly with an increase in the turbulence condition. For instance, the average BER decreases from $2.5\times 10^{-3}$ to $5\times 10^{-6}$ under weak turbulence, and the average capacity increases from 3.2 to 6.3 bps when the SNR is increased from 20 to 30 dB. However, the average BER decreases from $1.6\times 10^{-2}$ to $1.6\times 10^{-3}$ and average capacity increases from 1.3 to 2.9 bps under strong turbulence.

**Figure 3:**
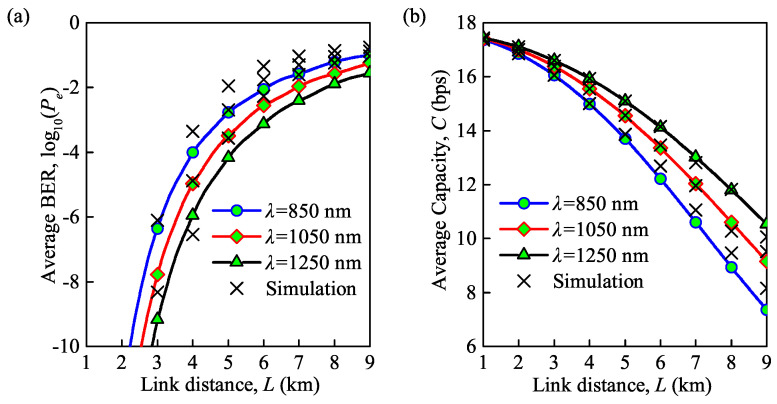
(**a**) Average BER versus link distance for different wavelengths; (**b**) average capacity versus link distance for different wavelengths ($C_{n}^{2}$ = $0.35\times 10^{-14}$$m^{-2/3}$ for weak turbulence, $T_{e}$ = 300 K, $R_{b}$ = 0.155 Gb/s).

Figure 3a,b demonstrate the effects of wavelength and link distance on the average BER and average channel capacity, respectively, under weak turbulence, for wavelengths of 850, 1050 and 1250 nm. Note that the average BER and average channel capacity in Figure 3a,b, respectively, are based on Equations (11) and (12) for the case of weak turbulence. It can be observed that the analytical and MC simulation results have a small deviation but can also validate the accuracy of the derived analytical expression for the average BER and average capacity. With an increase in the link distance *L*, the average BER increases and the average capacity decreases. This can be explained that based on the expression $\sigma_{x}^{2}=1.23C_{n}^{2}k^{\frac{7}{6}}L^{\frac{11}{6}}$, in which the link distance *L* is directly correlated with the log irradiance variance $\sigma_{x}^{2}$. Note that the log irradiance variance $\sigma_{x}^{2}$ is thereby related to the average BER and average capacity which have been expressed as Equations (11) and (12).

The average BER becomes smaller with an increase in the wavelength, while the average capacity becomes higher during this period. Note that the average capacity decreases suddenly while that of the average BER becomes slow when the link distance is very large. For instance, for λ = 850 nm, the average BER increases from $4.3\times 10^{-7}$ to $10^{-4}$ when the link distance is increased from 3 to 4 km, and the average capacity decreases from 16.1 to 15.0 bps. However, the average BER increases from $2.7\times 10^{-2}$ to $6.3\times 10^{-2}$, and average capacity decreases from 8.9 to 7.4 bps when the link distance is increased from 7 to 8 km.

**Figure 4:**
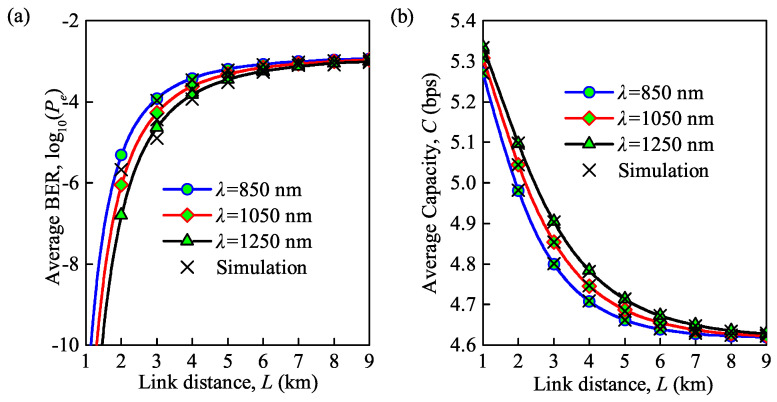
(**a**) Average BER versus link distance for different wavelengths; (**b**) average capacity versus link distance for different wavelengths ($C_{n}^{2}$ = $0.75\times 10^{-14}$$m^{-2/3}$ for moderate turbulence, $T_{e}$ = 300 K, $R_{b}$ = 0.155 Gb/s).

Based on Equations (16) and (19), the effects of the wavelength and the link distance on the average BER and average channel capacity under moderate turbulence are presented in Figure 4a,b. The link distance *L* is directly correlated with the log irradiance variance $\sigma_{x}^{2}$, and further results in the variation of the effective turbulence numbers at small scales α and large scales β in Equation (6). Note that the α and β are thereby related to the average BER and average capacity which have been expressed as Equations (16) and (19). The wavelengths are 850, 1050 and 1250 nm, respectively, and the overall trend of the change agrees with that shown in Figure 3a,b, although the average BER under moderate turbulence is larger than that under weak turbulence, and the corresponding channel capacity is about three times smaller than for weak turbulence. Besides, the analytical and MC simulation results have an exact match to validate the accuracy.

**Figure 5:**
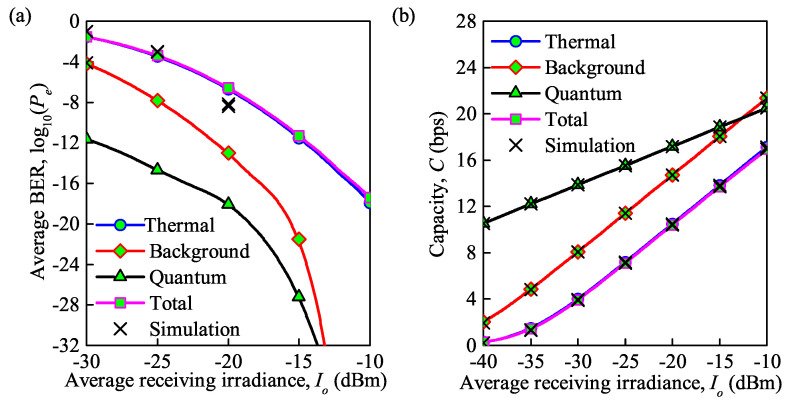
(**a**) Average BER versus average irradiance received under different types of noise; (**b**) average capacity versus average irradiance received under different types of noise ($C_{n}^{2}$ = $0.35\times 10^{-14}$$m^{-2/3}$ for weak turbulence, λ = 850 nm, $R_{b}$ = 0.155 Gb/s).

As shown in Figure 5a,b, different types of noise result in significant effects on the average BER and average channel capacity under weak turbulence. We first insert different noises, including total noise, background noise, thermal noise, and quantum noise, which can be expressed as $\sigma_{bg}^{2}+\sigma_{T_{e}}^{2}+\sigma_{Quantum}^{2}$, $\sigma_{bg}^{2}$, $\sigma_{T_{e}}^{2}$, $\sigma_{Quantum}^{2}$, into normalized SNR γ, as $\gamma =\frac{R^{2}A^{2}}{\sigma^{2}}$. Then, substituting γ into Equations (11) and (12), the average BER and average channel capacity are depicted in Figure 5a,b. Note that the above-mentioned forms of noise can also be expressed as, $\sigma_{bg}^{2}=\frac{2qI_{bg}R_{b}}{RI_{o}^{2}}$, $\sigma_{T_{e}}^{2}=\frac{4k_{1}T_{e}R_{b}}{RR_{L}I_{o}^{2}}$, $\sigma_{Quantum}^{2}=\frac{2qR_{b}}{RI_{o}}$. According to the expressions of various noises, we aim to discuss the influence of average irradiance received $I_{o}$. We can see that the analytical results provide a perfect match to the simulation results on average capacity, while considering average BER there is a deviation. On the other hand, for quantum noise, background noise, thermal noise and total noise, the average BER decreases and the average capacity increases with an increase in the average irradiance received.

The influence of the thermal noise on the average BER and average capacity is dominant. For instance, for $I_{o}$ = −20 dBm, the average BER for the total noise is $2.5\times 10^{-7}$ and the average capacity is 10.5 bps. When only thermal noise is considered, the average BER is $2\times 10^{-7}$ and the average capacity is 10.4 bps, while with only background noise, the average BER and average capacity are only $10^{-13}$ and 14.7 bps. Note that the same results can also be obtained in [28], which further certifies our theoretical derivation based on [30]. Besides, the impact of quantum noise is minimal; for example, the average BER and average capacity are only $7.9\times 10^{-19}$ and 17.2 bps when quantum noise is considered.

**Figure 6:**
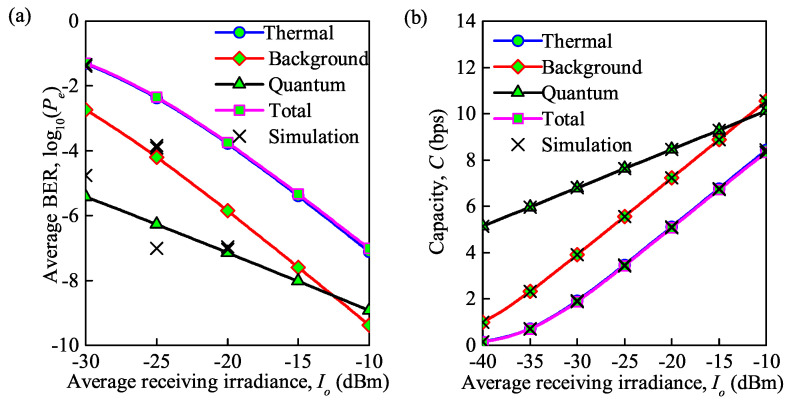
(**a**) Average BER versus average irradiance received under different types of noise; (**b**) average capacity versus average irradiance received under different types of noise ($C_{n}^{2}$ = $0.75\times 10^{-14}$$m^{-2/3}$ for moderate turbulence, λ = 850 nm, $R_{b}$ = 0.155 Gb/s).

Similar to the analysis of Figure 5, by substituting the expressions of different noises into Equations (16) and (19), the average BER and average channel capacity under moderate turbulence are demonstrated in Figure 6a,b, respectively. Note that the trends and the accuracy between analytical and MC simulation results in these graphs agree with those in Figure 5a,b. The specific values of the average BER and average channel capacity for the FSO communication system under different levels of channel turbulence and various types of noise at $I_{o}$ = −20 dBm are shown in Table 2. It is clear that the average capacity under weak turbulence is about twice that under a moderate regime, and the average BER is far lower than that under a moderate regime, when various types of noise are considered. For example, when only background noise is considered, the average BER and average capacity are $9.5\times 10^{-14}$ and 14.72 bps under weak turbulence, respectively, while under moderate turbulence, the average BER and average capacity are $1.4\times 10^{-6}$ and 7.22 bps.

As analyzed above, the thermal noise has a more obvious influence on the average BER and average channel capacity than the background noise and the quantum noise. Therefore, only the influence of the thermal noise on the system performance, including the average BER and average capacity, is further studied. Note that the thermal noise is correlated with several parameters, including temperature $T_{e}$, received irradiance $I_{o}$, and symbol transmission rates $R_{b}$ based on the expression $\sigma_{T_{e}}^{2}=\frac{4k_{1}T_{e}R_{b}}{RR_{L}I_{o}^{2}}$.

**Figure 7:**
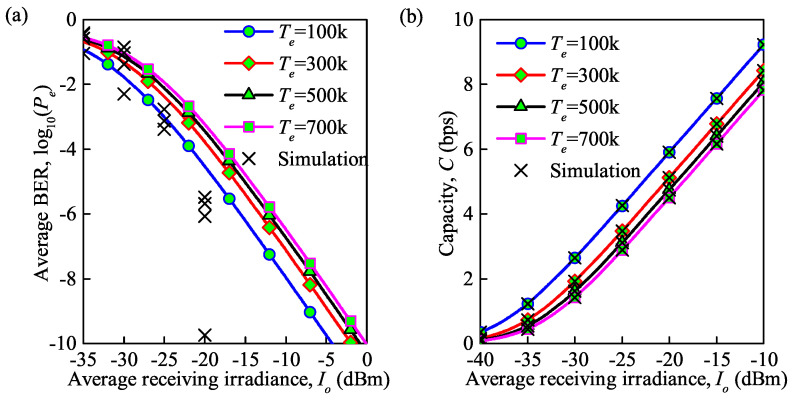
(**a**) Average BER at different temperatures under the influence of thermal noise; (**b**) average capacity at different temperatures under the influence of thermal noise ($C_{n}^{2}$ = $0.75\times 10^{-14}$$m^{-2/3}$ for moderate turbulence, λ = 850 nm, $R_{b}$ = 0.155 Gb/s).

The influence of the temperature on the average BER and average capacity are shown in Figure 7a,b, respectively. It can be observed that the average BER increases and the average capacity decreases with an increase in the temperature. Note that the variation tendency slows down during this period. At $I_{o}$ = −20 dBm, the increase in the average BER is 0.5 and the decrease in the average capacity is 0.24 bps when the temperature is increased from 500 to 700 K. However, the average BER increases by 0.25 and the average capacity decreases by 0.74 bps when the temperature is increased from 100 to 300 K. Theoretically, the influence of temperature on the FSO communication system is reasonable, since the structure function, $C_{n}^{2}$, of the atmospheric turbulence varies when the temperature fluctuates, which results in fluctuation in the refractive index. From a visual point of view, this fluctuation is reflected in the distortion of the picture. Furthermore, the strength of the atmospheric turbulence is represented by the variance in the refractive index fluctuation. As a result, atmospheric turbulence will lead to changes in the BER and capacity. The analytical and MC simulation results coincide which is clearly seen in the figure about average capacity, however, about average BER the results aloof.

**Figure 8:**
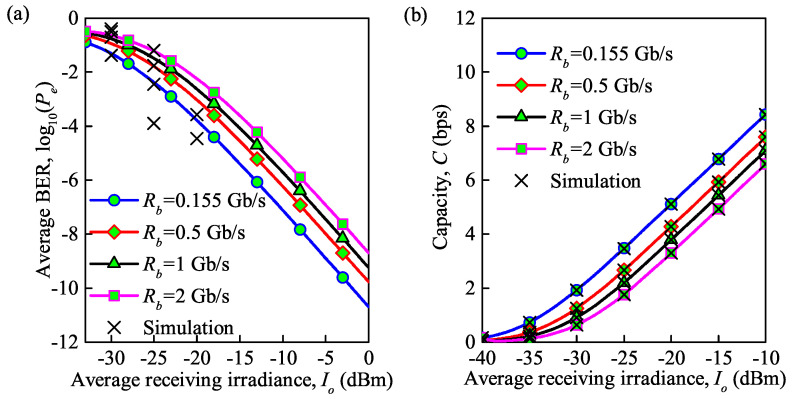
(**a**) Average BER for different symbol transmission rates under the influence of thermal noise; (**b**) average capacity for different symbol transmission rates under the influence of thermal noise ($C_{n}^{2}$ = $0.75\times 10^{-14}$$m^{-2/3}$ for moderate turbulence, λ = 850 nm, $T_{e}$ = 300 K).

In addition to the temperature, the symbol transmission rate also affects the variation in the average BER and average channel capacity. Figure 8 shows the performance in terms of the average BER and average capacity with an increase in the average received irradiance under moderate turbulence at different symbol transmission rates. It is clearly seen that average BER analytical results are not in a perfect agreement with the simulation results while average capacity analytical results are in an agreement with simulation results. It is interesting to observe that the trend is almost the same as in Figure 7. For example, at $R_{b}$ = 0.155 Gb/s, when the received irradiance increases from −30 to −20 dBm, the average BER decreases from $5\times 10^{-2}$ to $1.6\times 10^{-4}$, and the average capacity increases from 1.9 to 5.4 bps. The average BER decreases from $1.6\times 10^{-4}$ to $7.9\times 10^{-8}$, and the average capacity increases from 5.4 to 8.1 bps as the received irradiance increases from −20 to −10 dBm. This can be explained based on the expression $\sigma_{T_{e}}^{2}=\frac{4k_{1}T_{e}R_{b}}{RR_{L}I_{o}^{2}}$, in which the thermal noise is directly correlated with the symbol transmission speed.

**Figure 9:**
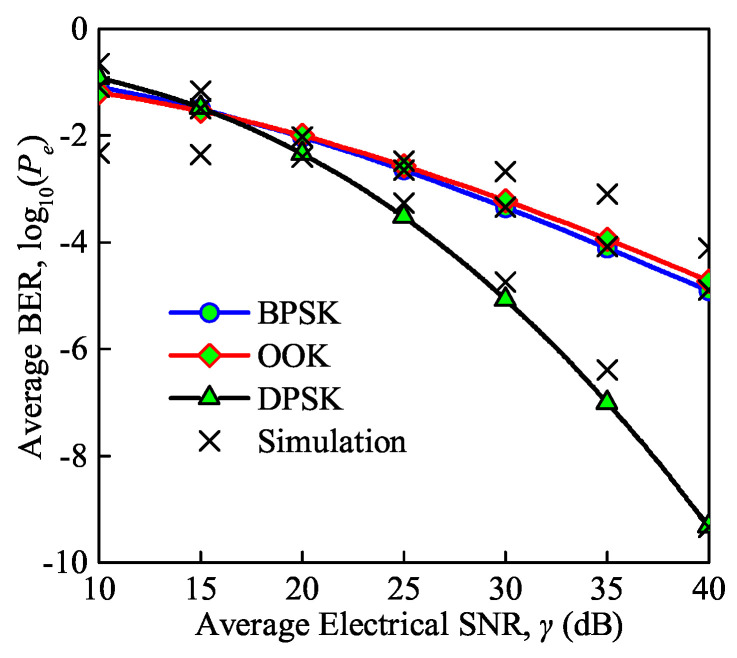
Average BER versus average electrical SNR under different modulation schemes ($C_{n}^{2}$ = $0.75\times 10^{-14}$$m^{-2/3}$ for moderate turbulence, λ = 850 nm, $T_{e}$ = 300 K, $R_{b}$ = 0.155 Gb/s).

The average BER versus the average electrical SNR under the BPSK, On-Off Keying (OOK) and Differential Phase Shift Keying (DPSK) modulation schemes for moderate turbulence is shown in Figure 9. Note that the background noise, thermal noise, and quantum noise are considered in this simulation as the total noise. We can conclude that with DPSK and BPSK modulation the analytical and MC simulation results have a good match validating the accuracy of the derived analytical expression for the average BER, while there is a small departure with OOK modulation. The average BER decreases gradually with an increase in the SNR. However, the average BER of the FSO communication system with DPSK modulation decreases more sharply than in the other modulation schemes. For instance, it can be observed that with DPSK modulation, the BER decreases from $5\times 10^{-3}$ to $7.9\times 10^{-6}$, while the average BER of the communication system with BPSK modulation and OOK modulation decreases from $10^{-2}$ to $5\times 10^{-4}$ and from $10^{-2}$ to $6.3\times 10^{-4}$, respectively, as the SNR increases from 20 to 30 dB. In addition, OOK modulation performs best when the SNR is less than 15 dB, while DPSK modulation performs best when the SNR is larger than 15 dB.

## 5. Conclusions

In this paper, the average BER and average capacity performance of an FSO communication system were investigated, taking into consideration the thermal, quantum and background noise. For the reason that lognormal and Gamma-Gamma distribution models have been verified to perform well for weak and moderate-to-strong turbulence in recent research, both these two models of atmospheric turbulence were employed and the system performance was compared under these models with different noises. It was found that both the system parameters (such as the transmission rate, wavelength, and link distance) and the channel parameters (such as the structure constant and temperature) play crucial roles in the scintillation index, further affecting the average BER and the average capacity performance of the FSO communication system. Simulation results also reveal that thermal noise has a more obvious influence on the system performance than the other types of noise. In addition, it was shown that OOK and DPSK modulation performed best in the lower and higher SNR regimes, respectively. Above all, the FSO system will perform better through adjusting parameters to restrain noises or improve turbulence and choosing appropriate modulations.

## Figures and Tables

**Table 1:** Parameters used in the simulations.

Symbol	Description	Value (Unit)
*R*	Photodetector responsivity	1
$R_{L}$	Receiver circuit load resistance	$50\;\left(\Omega \right)$
$T_{e}$	Temperature	$300\;\left(K\right)$
*q*	Elementary charge	$1.602\times 10^{-19}\;\left(C\right)$
$k_{1}$	Boltzmann constant	$1.38\times 10^{-23}\;\left(J/K\right)$
$R_{b}$	Symbol rate	$1.55\times 10^{8}\;\left(\mathrm{bps}\right)$
*B*	Bandwidth	$10^{-3}\;\left(\mathrm{bit}/s\right)$
$I_{bg}$	Background radiation irradiance	$\frac{4}{\pi }\times 0.6^{2}\times 10^{-6}+\;5.5\times 10^{-5}\;\left(A\right)$

**Table 2:** Average BER and average channel capacity of an FSO communication system under weak and moderate turbulence conditions with various types of noise ($I_{o}$ = −20 dBm).

Turbulence Conditions	Performance	Thermal Noise	Background Noise	Quantum Noise	Total Noise
Weak	Average BER	$2\times 10^{7}$	$9.5\times 10^{-14}$	$8.5\times 10^{-19}$	$2.5\times 10^{-7}$
	Average capacity (bps)	10.50	14.72	17.19	10.41
Moderate	Average BER	$1.7\times 10^{-4}$	$1.4\times 10^{-6}$	$7.2\times 10^{-8}$	$1.8\times 10^{-4}$
	Average capacity (bps)	5.11	7.22	8.46	5.07

## Data Availability

Not applicable.

## Appendix A. Definition of Meijer’s G Function

The Meijer’G function is defined as [29],

(A1) $$\begin{array}{cc} G_{p,q}^{m,n}\left(z\left|\begin{array}{c} a_{1},\ldots ,a_{n},a_{n+1},\ldots ,a_{p}\\ b_{1},\ldots ,b_{m},b_{m+1},\ldots ,b_{q} \end{array}\right.\right) & =\frac{1}{2\pi i}\int_{L}\frac{\prod_{k=1}^{m}\Gamma \left(s+b_{k}\right)}{\prod_{k=n+1}^{p}\Gamma \left(s+a_{k}\right)}\\ \; & \cdot \frac{\prod_{k=1}^{n}\Gamma \left(1-s-a_{k}\right)}{\prod_{k=m+1}^{q}\Gamma \left(1-s-b_{k}\right)}z^{-s}ds, \end{array}$$

where m∈N∧n∈N∧p∈N∧q∈N∧m≤q∧n≤p, and *L* is the infinite contour of integration.
